# Acute pancreatitis in a COVID‐19 patient: An unusual presentation

**DOI:** 10.1002/ccr3.3412

**Published:** 2020-10-27

**Authors:** Goutam Kumar Acherjya, Md Masudur Rahman, Mohammad Touhidul Islam, ABM Saiful Alam, Keya Tarafder, Mohammad Mostafizur Rahman, Mohammad Ali, Shudip Ranjan Deb

**Affiliations:** ^1^ Medicine Jashore Medical College Jashore Bangladesh; ^2^ Respiratory Medicine Jashore Medical College Jashore Bangladesh; ^3^ Cardiology 250 Bedded General Hospital Jashore Bangladesh; ^4^ Microbiology Jashore Medical College Jashore Bangladesh; ^5^ Haematology National Institute of Cancer Research and Hospital Dhaka Bangladesh; ^6^ Medicine Mugda Medical College Dhaka Bangladesh

**Keywords:** Acute Pancreatitis, Atypical Presentations, COVID‐19, SARS‐CoV‐2

## Abstract

Any atypical presentation of COVID‐19 may be occurred as a part of its elevated coagulopathy or cytokine storm syndrome. So therefore, physicians should be aware and prepared to handle such atypical presentations and sequelae related to COVID‐19.

## Graphical abstract

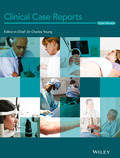

## 1 BACKGROUND

The entire world has been crippled by the severe acute respiratory syndrome (SARS‐CoV‐2) virus infection starting mid‐December, 2019. SARS‐CoV‐2 is a novel new virus, and there was scarcity of established data regarding its nature, clinical manifestations, and treatment protocol and outcomes. Initially, coronavirus disease‐2019 (COVID‐19) caused by SARS‐CoV‐2 virus is thought to be manifested by respiratory illness such as cough, chest tightness, and dyspnea. Over time, atypical presentations including cardiac, hepatic, renal, musculoskeletal, gastrointestinal, and neurological features have been identified by COVID‐19. Therefore, we are presenting a confirmed case of COVID‐19 who developed acute pancreatitis during acute disease phase without any other obvious discernible cause of it.

A series of pneumonia had been identified by a homological virus similar to severe respiratory syndrome coronavirus (SARS‐CoV) which was classified as a novel coronavirus‐19, later on classified as SARS‐CoV‐2 virus and the disease as COVID‐19 in Wuhan city, Hubei province, China in December 2019.^1^, ^2^ Very soon, the new contagious virus spread all over the world including 213 countries and territories with an estimation of more than 15 million total confirmed SARS‐CoV‐2 case and six hundred thousand people died of COVID‐19 according to the live update of Worldometer by 22 July 2020.^3^ Initially, COVID‐19 is manifested by respiratory symptoms including fever, dry cough, dyspnea, pharyngodynia, nasal congestion, and rhinorrhea.^4^ Gradually, a lot of atypical presentations including various gastrointestinal symptoms, ageusia, anosmia, infarction or hemorrhagic stroke, Guillain‐Barré syndrome, acute necrotizing encephalopathy, cardiac arrhythmias, pericarditis, myocarditis, heart failure, pulmonary embolism, deep venous or arterial thrombosis, acute kidney injury may occur even without prior respiratory symptoms.^5^, ^6^ However It is strongly believed that the severity with multi‐organ failure of COVID‐19 results as a part of cytokine release syndrome or cytokine storm and hypercoagulability.^7^, ^8^ Hereby, we are reporting an atypical case who developed acute pancreatitis during the disease process of COVID‐19 without any precipitating causes.

## 2 CASE PRESENTATION

**Figure 1:**
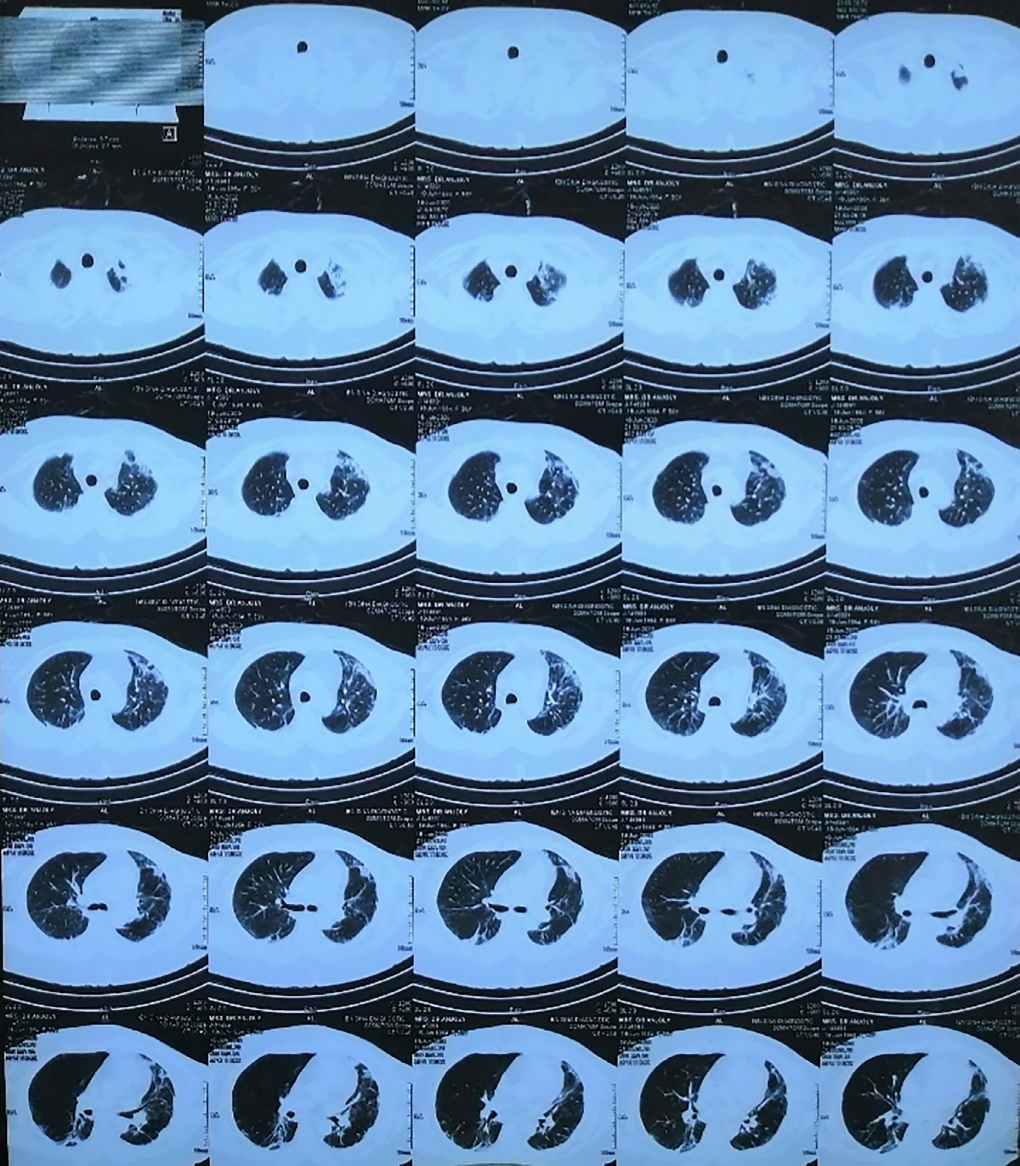
HRCT of chest

**Figure 2:**
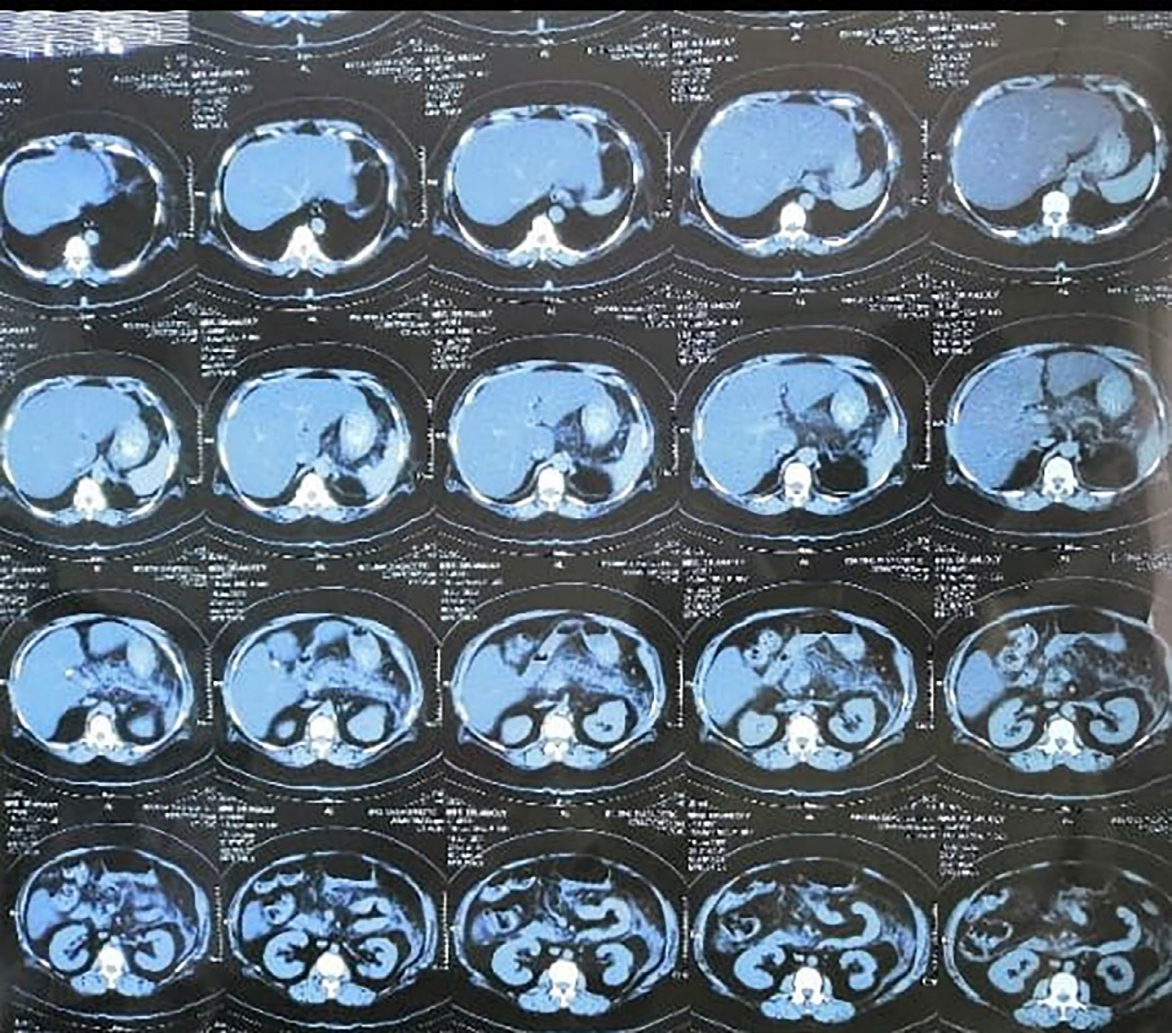
CT scan of Abdomen

A 57‐year‐old female physician with previous history of hypertension, type 2 diabetes mellitus and active malignancy of breast and larynx developed high grade fever, generalized body ache, loss of smell, fatigue, and arthralgia for past two days. Before getting these new symptoms, she had been receiving radiotherapy, trastuzumab, losartan, metformin, and premix insulin as per physician's schedule. On the basis of the high indexed symptoms of COVID‐19, real‐time polymerase chain reaction (RT‐PCR) was ordered which had returned positive. We had found high CRP and serum ferritin; and mild‐to‐moderate involvement of both lungs having diffuse ground glass opacities with crazy‐paving pattern with small consolidation in the chest CT scan. (Table 1, Figure 1) Acquisition of confirmed COVID‐19 report, she was prescribed standard dose of favipiravir (1600 mg twelve hourly on the first day followed by 600mg twelve hourly on the subsequent day) and prophylactic dose of Enoxaparin 40 IU (International Unit) daily. She remained clinically stable with remission of fever and improvement of her symptoms including her oxygen saturation maintained 95%‐97% on room air for next two days. On the 5th day after symptom onset, her oxygen saturation was down to 87% on the room air which made her admitted in a local private hospital in Jashore, Bangladesh. Shortly after oxygen inhalation at a rate of 4 liters/minute by nasal cannula were started and her oxygen saturation were maintained at 98%. On the next day, her oxygen saturation was maintained at 96%‐97% on the room air and she felt eventually well during the daytime. She developed mild epigastric pain without any other lateralizing sign and symptoms which was relieved by supportive treatment. On the 9th day of her COVID‐19 positive, she again developed severe epigastric pain radiating to the back with vomiting for two times. Clinical examination revealed upper abdominal tenderness with presence of bowel sound. Both clinical signs and symptoms suggested high index of suspicion for acute pancreatitis. She has, of note, no history of alcohol use, recent abdominal or cardiopulmonary surgery, abdominal trauma or use of any known offending drugs that may cause acute pancreatitis. Therefore, we had suggested investigation including complete blood count, C‐reactive protein, liver function test, serum creatinine, serum electrolytes, serum lipase, D‐dimer, and CT scan of abdomen. Finally, she was diagnosed as a case of acute pancreatitis on the basis of high serum Lipase (8352 U/L) and abdominal CT scan (moderately swollen pancreas) according to the Atlanta classification and definition by international consensus.(Table 2, Figure 2).

**Table 1:** Report of base line investigations following COVID‐19 positive

Serial Number	Investigations	Results
01	CBC	TC: 3000/cmm, (N‐68%, L‐24%), ESR‐mm in 1st hour, Hemoglobin‐12.7 gm/dL, Platelet count‐ 148 000/cmm.
02	C‐Reactive Protein	25.2 mg/L
03	Fasting blood glucose	13.84 mmol/L.
04	SGPT	54 U/L.
05	Serum Creatinine	1.00 mg/dL
06	HbA1c	8.3%.
07	D‐Dimer	200 mcg/L. (Reference value‐ less than 200mcg/L)
08	Serum Ferritin	397 ng/mL. (Reference value‐ less than 186ng/ml)
09	ECG	Normal limit.
10	Chest Radiography	Ill‐defined patchy opacity in the right lower zone.
11	CT Scan Chest	Mild‐to‐moderate involvement of both lungs having diffuse glass opacities with crazy‐paving pattern, small consolidation, and fibrotic strands.

**Table 2:** Laboratory findings following development of acute pancreatitis

Serial Number	Investigations	Results
01	CBC	TC‐8730/cmm, (N‐87%, L‐09%), Hemoglobin‐13.5g/dl, Platelet count‐75000/Cmm
02	Routine Urine	Albumin (+), Glucose (++), Leukocyte (+), and pus cells‐ 12‐15/HPF
03	Postprandial blood sugar	17.89 mmol/L
04	C‐Reactive Protein	233mg/L
05	Serum Amylase	80 U/L. (Reference value‐ less than 115U/L)
06	Serum Lipase	8352 U/L. (Reference value‐ less than 300U/L)
07	Prothrombin time	21.0 second (Control‐ 13.0 second, INR‐1.64)
08	Serum Albumin	2.82 gm/dL
09	Serum Creatinine	0.79 mg/dL
10	Serum BUN	12.14 mg/dL
11	Serum Calcium	5.50 mg/dL
12	Serum Electrolytes	Na‐ 142 mmol/L, K‐ 3.80mmol/L, Cl‐ 105 mmol/L, HCO2‐ 21 mmol/L.
13	SGPT	25 U/L
14	D‐Dimer	800 mcg/L. (Reference value‐ less than 200 mcg/L)
15	Fasting Lipid Profile	Tc‐134 mg/dL, HDL‐ 23 mg/dl, LDL‐55.80 mg/dL, Tg‐ 276 mg/dL
16	ECG	Within normal
17	CT Scan of whole abdomen	Mildly swollen and inflamed pancreas, postcholecystectomy status and fatty liver.

## 3 INVESTIGATION

We had obtained baseline investigations after her diagnosis as COVID‐19 including CBC, Random blood sugar, S. Creatinine, SGPT, CRP, D‐Dimer, S, Ferritin, HbA1c, S. electrolytes, prothrombin time with INR (international normalization ratio), and chest radiography. (Table 1) Prognostic features of acute pancreatitis had extensively evaluated including S. Albumin and S. Calcium. Fasting lipid profile was measure to see the triglyceride level for exclusion of precipitating factor.(Table 2).

## 4 TREATMENT

With this above scenario of acute pancreatitis she was treated with adequate bowel rest, intravenous fluid therapy, injectable broad‐spectrum antibiotics, omeprazole, and pethidine hydrochloride. Our expert opinion suggested, in the absence of confirmatory data, to stop favipiravir in light of new molecule and temporal association with acute pancreatitis in this case. Gradually, we noticed her uneventful improvement on current medications including stable oxygen saturation 97% on room air. Finally, she was discharged on oral medications with symptoms free from acute pancreatitis after testing negative RT‐PCR for COVID‐19. We advised her to continue injection Enoxaparin at a therapeutic dose for the next 14 days at home followed by oral rivaroxaban 10mg daily. Her ongoing antihypertensive and antidiabetic medications Losartan Potassium and insulin were continued, respectively, whereas Metformin was withheld shortly after her positive RT‐PCR test of COVID‐19.

## 5 OUTCOME AND FOLLOW‐UP

**Figure 3:**
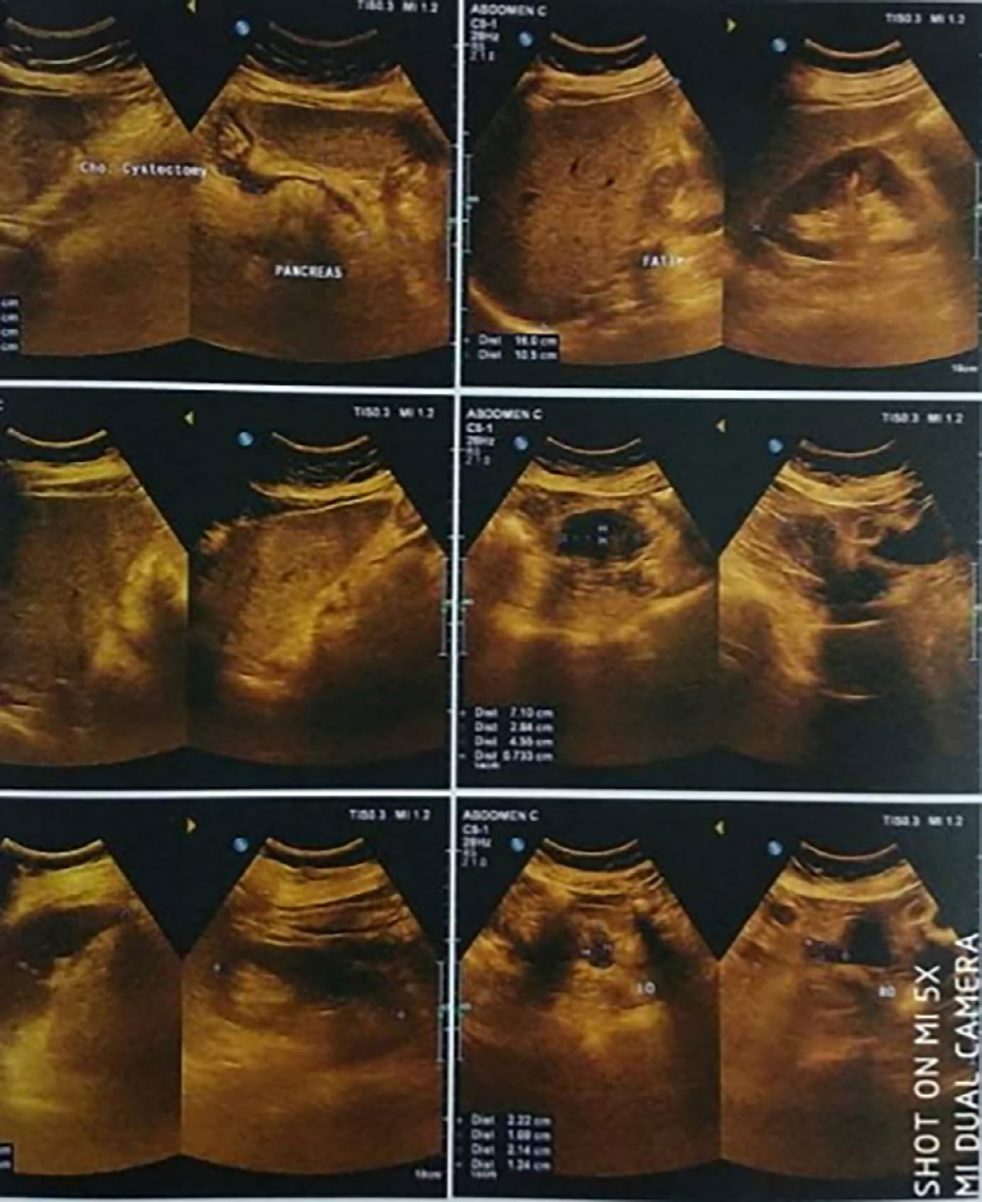
Follow‐up Ultrasonography of Abdomen

**Figure 4:**
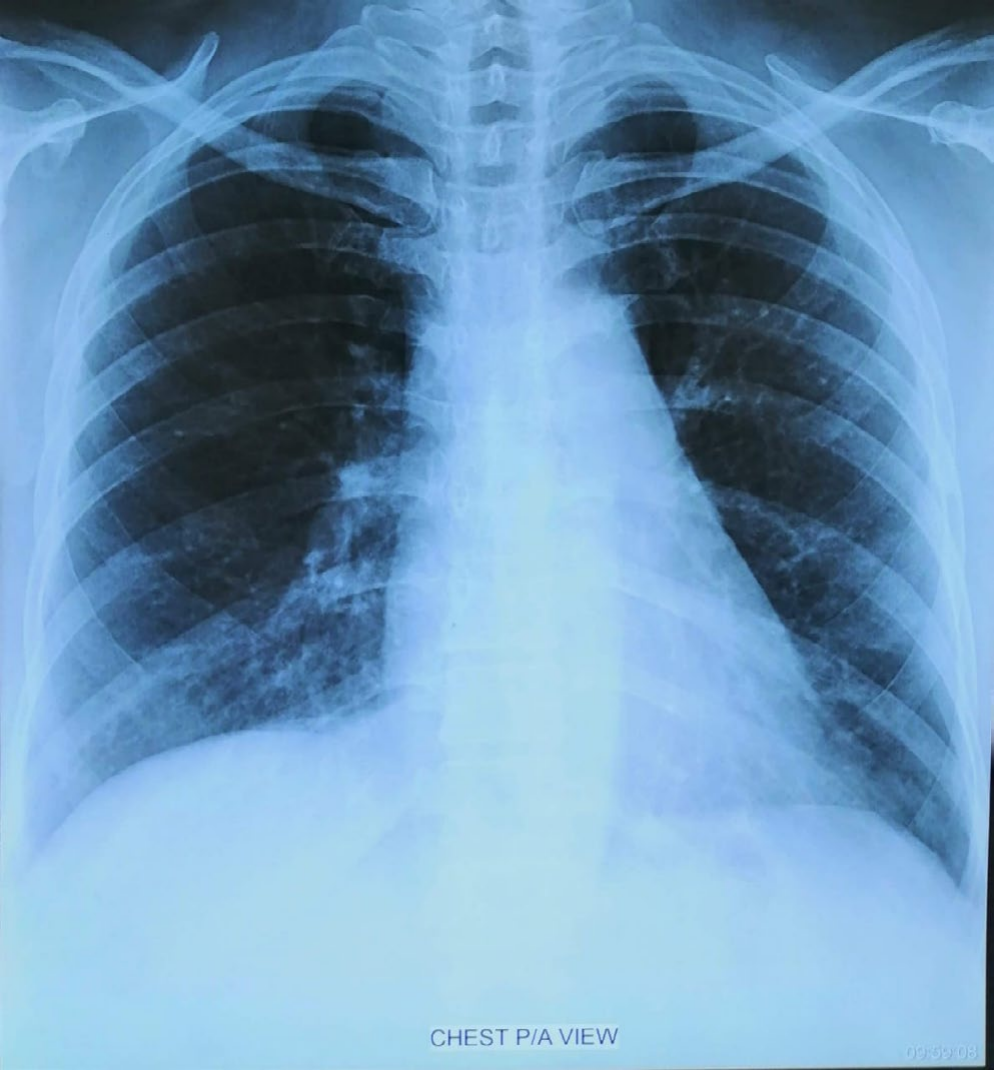
Follow‐up Chest X‐Ray P/A view

However, her discharge period was also uneventful and we had followed her up 7 and 14 days later with complete blood count, C‐reactive protein, random blood sugar, liver function test, S. creatinine, S. Lipase, S. electrolytes, D‐Dimer, chest radiography, and ultrasonography of whole abdomen (Tables 3 and 4, Figures 3 and 4). She had not developed any secondary complications of acute pancreatitis during her discharge period.

**Table 3:** Results of first follow‐up investigation following 7 days of acute pancreatitis

Serial Number	Investigations	Results
01	CBC:	TC‐6540, (N‐88%, L‐08%), ESR‐104mm in 1st hour, Hemoglobin‐10.9 gm/dL, Platelet Count‐210 000/Cmm
02	PPBS	9.23 mmol/L
03	C‐Reactive Protein	163 mg/L
04	S. Creatinine	0.63 mg/dL
05	S Albumin	3.02 g/dL
06	Serum Calcium	7.70 mg/dL
07	Serum Lipase	422U/L. ()
08	Prothrombin Time	15 s (Control‐13, INR‐1.16)
09	S electrolytes	Na‐ 138 mmol/L, K‐ 4.00 mmol/L, Cl‐ 102 mmol/L, HCO2‐29
10	D‐Dimer	400 mcg/L. (Reference value‐ less than 200 mcg/L)

**Table 4:** Results of second follow‐up investigations following 14 days of acute pancreatitis

Serial Number	Investigations	Results
01	CBC	TC‐7780/Cmm, (N‐77%, L‐12%), ESR‐84mm in 1st hour, Hemoglobin‐11.0 g/Dl, Platelet Count‐283 000/Cmm
02	RBS	12.22 mmol/L
03	C‐Reactive Protein	23.1 mg/L
04	S. Creatinine	0.83 mg/dL
05	S Bilirubin	0.33 mg/dL
06	ALT/SGPT	17 U/L
07	AST/SGOT	26 U/L
08	Alkaline Phosphatase	110 U/L
09	D‐Dimer	400 mcg/L. (Reference value‐ less than 200 mcg/L)
10	S Electrolytes	Na‐ 140 mmol/L, K‐2.60 mmol/L, Cl‐97 mmol/L, HCO2‐31 mmol/L
11	Chest Radiography	Ill‐defined patchy opacity in the right lower zone.
12	USG of whole abdomen	Suggestive of fatty liver.

## 6 DISCUSSION

According to the Atlanta classification and definition by international consensus, acute pancreatitis can be diagnosed following presence of at least two out of three points: (a) abdominal pain suggestive of acute pancreatitis, (b) lipase or amylase more than three times higher the normal upper limit, and (c) sonography or radiography findings pertinent with acute pancreatitis.^9^ Therefore, our case met the criteria and was diagnosed as an acute pancreatitis. Though the exact mechanism of acute pancreatitis by SARS‐CoV‐2 infection is unknown but the cytopathic effect, systemic inflammatory responses or harmful immune response mediated by local SARS‐CoV‐2 replication or infection are thought to be responsible for the pathogenesis of acute pancreatitis here. However, SARS‐CoV‐2‐mediated acute pancreatic injury had been evident^10^ but COVID‐19 induced established acute pancreatitis case has seldomly been reported till date globally.^6^, ^11^, ^12^ Moreover, there is no reported case of acute pancreatitis resulting from Favipiravir; and other medications taken earlier by our patient which is insignificantly related to it.^13^ So therefore, it is safe to assume that acute pancreatitis here in our patient developed as a part of COVID‐19.

## 7 CONCLUSION

Wide spectrum atypical presentations including acute pancreatitis are evident by SARS‐CoV‐2 infection. Therefore, any manifestation of its complication should be kept as high index clinical suspicious during management of COVID‐19.

## CONFLICT OF INTEREST

None declared.

## AUTHOR CONTRIBUTIONS

GKA: designed concept, interpreted data, reviewed literature, drafted, and gave critical review of this manuscript. KT and MA: were involved in drafting and literature review of this manuscript. MR, TR, ABMSA, and MR: were engaged in collecting data and literature review. SRD: had given critical review for the manuscript.

## PATIENT CONSENT FOR PUBLICATION

Obtained.
